# Accuracy of MRI Versus PET/CT in the Prediction of Treatment Response to Neoadjuvant Chemotherapy in Breast Cancer

**DOI:** 10.7759/cureus.66114

**Published:** 2024-08-04

**Authors:** Noof Alshaibani, Janaki Krithika Chandramohan, Yusuf Althawadi, Maryam Almusalam, Sara S Khairi, Hamad S Saif, Khalid Al Sindi, Salwa Aly

**Affiliations:** 1 Department of Oncoplastic and Reconstructive Breast Surgery, Bahrain Oncology Center, Muharraq, BHR; 2 Department of Pathology, King Hamad University Hospital, Al Sayh, BHR; 3 Department of Nuclear Medicine, King Hamad University Hospital, Al Sayh, BHR

**Keywords:** neoadjuvant chemotherapy, pet-ct scan, general surgery breast cancer, breast cancer outcomes, mri breast, breast cancers

## Abstract

Background

Breast-conserving surgeries have significantly advanced breast cancer treatment, offering favorable oncological outcomes, enhanced cosmetic results, reduced postoperative morbidity, and better psychological acceptance compared to mastectomy. The introduction of neoadjuvant therapy has expanded the applicability of breast conservation surgery to include locally advanced tumors. Tumor response to neoadjuvant chemotherapy is evaluated using imaging modalities such as breast ultrasound, breast magnetic resonance imaging (MRI), and positron emission tomography/computed tomography (PET/CT). Accurate prediction of therapeutic response facilitates the planning of surgical and adjuvant treatments. This study aims to compare the diagnostic accuracy of MRI and PET/CT in predicting treatment response to neoadjuvant chemotherapy in breast cancer patients.

Methods

This retrospective study was conducted at a tertiary care center in Bahrain. A total of 138 patients with locally advanced breast cancer or human epidermal growth factor receptor-2 (HER2) positive, hormone receptor-negative cancers who underwent breast-conserving surgeries between June 2018 and December 2022 were included. The inclusion criteria focused on patients achieving a complete pathological response following neoadjuvant systemic therapy, ensuring a homogenous study population. Patients with hormone receptor-positive early breast cancers or metastatic tumors, ineligible for neoadjuvant chemotherapy, were excluded. Non-responders and partial responders were also excluded from the study. Statistical analysis was performed using IBM SPSS v26.0 (IBM Corp., Armonk, US). Response rates for the imaging modalities and histopathology results were assessed. Agreement between histology and imaging modalities was computed using kappa statistics. Diagnostic performance for predicting "no residual" disease was evaluated using the McNemar Test. All tests were two-tailed, with a p-value <0.05 considered statistically significant.

Results

The study included 138 patients, of whom 73 (52.9%) had an incomplete response or residual disease, while 65 (47.1%) had a complete response or no residual disease according to histology reports. There was slight agreement between post-neoadjuvant MRI and histology results (Cohen’s kappa 0.172, p=0.010), while substantial agreement was observed between post-neoadjuvant PET/CT and histology results (Cohen’s kappa 0.614, p=0.000). PET/CT demonstrated a higher sensitivity of 93.8% (p<0.001) and a specificity of 68.5%. Although MRI was more specific, the positive predictive value was comparable for both PET/CT and MRI.

Conclusion

PET/CT shows higher sensitivity and can serve as an early marker for predicting complete pathological response in post-neoadjuvant breast cancer patients. However, the prediction of residual disease is optimized by combining both MRI and PET/CT as diagnostic modalities.

## Introduction

Breast cancer is the leading cause of cancer-related deaths in females all over the world. Recently, mortality rates have decreased due to screening mammography and the new screening approaches [1]. For patients with locally advanced breast cancer (LABC), neoadjuvant chemotherapy (NAC) followed by surgery is the main treatment option [2]. Pathologic complete response (pCR) is the ultimate outcome for NAC, and it is an important prognostic factor for both disease-free survival and overall survival of breast cancer [3]. Thus, early response assessment and accurate prediction of pCR to NAC are crucial because they can help determine the size of the residual tumor before surgery [4]. This can lead to more precise surgical resection, more preservation of breast tissue, and fewer chances of positive margins. After NAC, patients with breast cancer are evaluated using different diagnostic methods, such as physical examination, breast imaging with mammography/ultrasonography or magnetic resonance imaging (MRI), scanning the body with positron emission tomography/computed tomography (PET/CT), and examining the tissue samples. On one hand, MRI is becoming more popular and recommended as a precise imaging technique for evaluating NAC response in patients with operable breast cancer [5,6]. MRI uses the size and microvasculature of the breast tumor to predict pCR [7,8]. 

Although MRI is becoming more common and suggested as an accurate imaging method [7], MRI imaging may have drawbacks, such as it cannot tell apart living tumor tissue from scar tissue made of fibrous tissue [8]. On the other hand, 18F-fluoro-2-deoxyglucose (FDG) PET/CT imaging has an established role in predicting pCR. FDG PET/CT uses radioactive glucose to show the activity of cells in evaluating the tumor response early on [9]. FDG PET/CT imaging can measure the changes in the energy use of tumors, which happen during the course of NAC. However, these energy use indicators may not always match the MRI parameters that use contrast dye, which is affected by regional blood flow [10]. Furthermore, while studies such as those conducted by Sheikhbahaei et al. and Liu et al. have contributed significantly to our understanding of the predictive accuracy of MRI and PET/CT individually [11,12], there is a distinct lack of head-to-head comparative analyses between these techniques. These studies, while informative, have primarily evaluated each modality's capabilities in isolation. Consequently, the precise comparative diagnostic performance of MRI and PET/CT in predicting treatment response remains relatively unexplored. Building on this foundation, our study aims to comprehensively evaluate and compare the diagnostic accuracy of MRI and PET/CT in predicting treatment response to neoadjuvant chemotherapy in locally advanced breast cancer patients.

By analyzing the correlation between these imaging modalities and postoperative histopathology reports, our research seeks to provide further insights into optimal imaging strategies for assessing neoadjuvant chemotherapy response. The outcomes of our study are anticipated to contribute significantly to the management of breast cancer, aiding clinicians in making informed decisions regarding treatment approaches and ultimately enhancing patient care.

## Materials and methods

Our retrospective study was conducted at the King Hamad University Hospital in Bahrain. The work was authorized by the institutional review board’s ethical committee. We included patients who had been diagnosed with locally advanced or hormone receptor-negative breast cancers. Patient data collection spanned a six-month duration, encompassing cases operated between June 2018 and December 2022. The study's inclusion criteria were centered around patients achieving a complete pathological response following neoadjuvant systemic therapy, ensuring a uniform and well-defined study population. A total of 138 patients with locally advanced breast cancer or human epidermal growth factor receptor-2 (HER2) positive, hormone receptor-negative cancers were screened. We excluded patients with hormone receptor-positive early breast cancers or metastatic tumors, who were ineligible for neoadjuvant chemotherapy. The non-responders and partial responders were identified and excluded from the study. pCR is defined as the disappearance of all invasive cancer in the breast after completion of neoadjuvant chemotherapy. Breast disease response to neoadjuvant therapy served as the primary inclusion criteria in our study. The final sample size of 65 patients, conforming to the above-mentioned complete pathological response following neoadjuvant therapy, was thus arrived at.

Sampling was facilitated by accessing patients' records in the hospital's electronic medical records, identifying those who had undergone surgery after neoadjuvant chemotherapy. Recruitment was contingent upon patients' neoadjuvant chemotherapy status while preserving the anonymity of patient demographics and focussing solely on pertinent investigations. 

To assemble our study cohort, we accessed the surgical list in the hospital's electronic medical record (HOPE) system. This list was consolidated and cross-referenced with the National Tumour Board documentation to track the neoadjuvant treatment journeys of the patients. Final pathological reports, crucial for identifying complete responders, were sourced from HOPE.

Subsequent to patient data compilation, a meticulous reanalysis phase was initiated involving a comprehensive comparison of pre- and post-neoadjuvant MRI and PET/CT reports. The central objective was to evaluate the predictive accuracy of the imaging modalities concerning complete pathological response. 

In terms of data management and analysis, the MRI and PET/CT data collected prior to and after neoadjuvant treatment were systematically organized into tabulated datasets. These datasets were then compared to the final postoperative histopathology report.

Statistical methods

IBM SPSS v26.0 (IBM Corp., Armonk, US) was used for statistical analysis. The response rates were assessed for imaging modalities and histopathology results. The agreement in responses of histology and imaging modalities was computed using Kappa statistics. The diagnostic performance in the prediction of “no residual” was done using the McNemar test. The receiver operating curve (ROC) method was used to generate the classification model for the diagnostics used in the study. The Youden index (YI) was used to determine the optimal cut-off. A confusion matrix to represent the accuracy of the model was generated using training data. All the tests were two-tailed and a p-value <0.05 was considered statistically significant.

## Results

A total of 138 patients were included in the study, of which 73 (52.9%) exhibited an incomplete response or residual disease, while 65 (47.1%) demonstrated a complete response or absence of residual disease according to histology reports. Table 1 presents the cross-tabulation of the imaging modalities against the histology findings. 

**Table 1:** Number of patients with complete and incomplete pathological response and their distribution as predicted using imaging techniques PET and MRI PET: Positron emission tomography; MRI: Magnetic resonance imaging

		Pathological response	
		Residual	No residual
Post-neoadjuvant MRI	Residual	66	48
	No residual	7	17
Post-neoadjuvant PET	Residual	50	4
	No residual	23	61
Total		73	65

**Figure 1:**
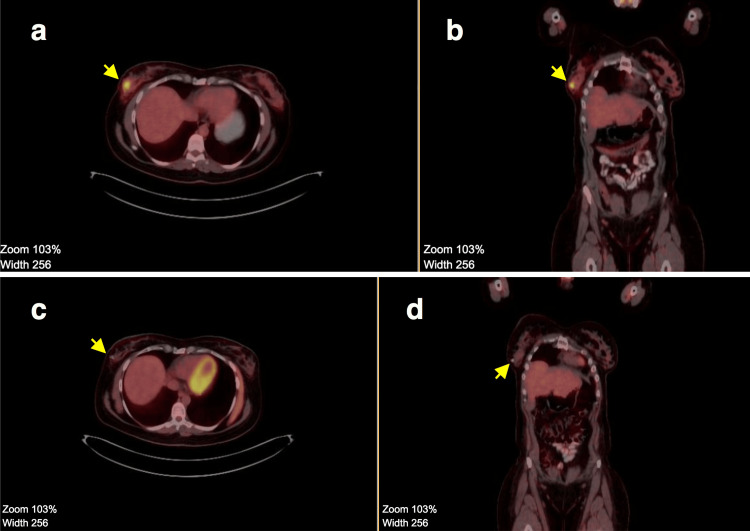
Comparison between baseline PET scan and post-neoadjuvant PET scan showing complete metabolic regression of the lesion (a) Baseline PET scan - axial section with an arrow pointing to the hypermetabolic lesion. (b) Baseline PET scan - coronal section with an arrow pointing to the hypermetabolic lesion. (c) Post-neoadjuvant PET scan - axial section with an arrow pointing to the clip (site of the lesion). (d) Post-neoadjuvant PET scan - coronal section with an arrow pointing to the clip (site of the lesion). PET: Positron emission tomography

**Figure 2:**
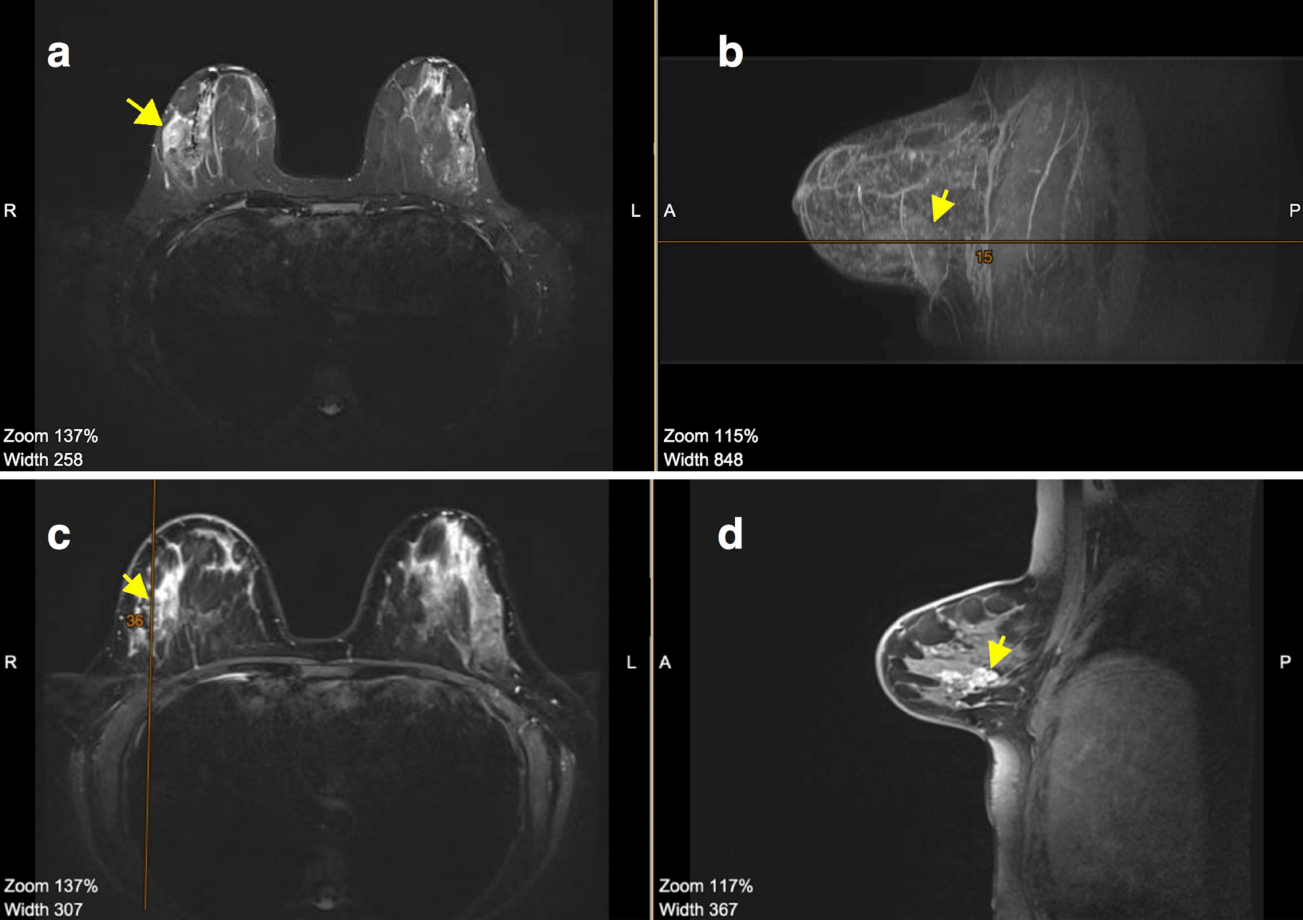
Comparison between baseline MRI and post-neoadjuvant MRI of the breasts showing partial regression of the lesion (a) Baseline MRI of the breast - axial section with an arrow pointing to the lesion. (b) Baseline MRI of the right breast - sagittal section with an arrow pointing to the lesion. (c) Post-neoadjuvant MRI of the breast - axial section with an arrow pointing to the lesion. (d) Post-neoadjuvant MRI of the right breast - sagittal section with an arrow pointing to the lesion. R: Right; L: Left; A: Anterior; P: Posterior; MRI: Magnetic resonance imaging

There was a slight level of agreement between post-neoadjuvant MRI and histology results (Cohen’s kappa 0.172, p=0.010), whereas there was a substantial significant level of agreement between post-neoadjuvant PET/CT and histology results (Cohen’s kappa 0.614, p=0.001) (Table 2). Figures 1-2 are representative figures showing PET/CT findings correlating with pathological complete response as opposed to MRI showing significant residual disease.

**Table 2:** Cohen's kappa agreement between the imaging techniques for pCR * p-value <0.05, which is significant MRI: Magnetic resonance imaging; PET: Positron emission tomography; pCR: Pathological complete response

Pathological complete response		
	Cohen’s kappa statistics	p-value
Post-neoadjuvant MRI	0.172	0.010*
Post-neoadjuvant PET	0.614	0.001*

The PET results showed a higher sensitivity for predicting the no residual (complete response) group's pathological results. The positive predictive value was, however, comparable for both PET and MRI (Table 3).

**Table 3:** The diagnostic performance of MRI and PET for the prediction of no residual tumor (complete pathological response) * p-value <0.05, which is significant MRI: Magnetic resonance imaging; PET: Positron emission tomography

Parameter	MRI	PET	p-value
Sensitivity (%)	26.2	93.8	0.001*
Specificity (%)	90.4	68.5	0.001*
Positive predictive value (%)	70.8	72.6	0.875
Negative predictive value (%)	57.9	92.6	0.001*

**Figure 3:**
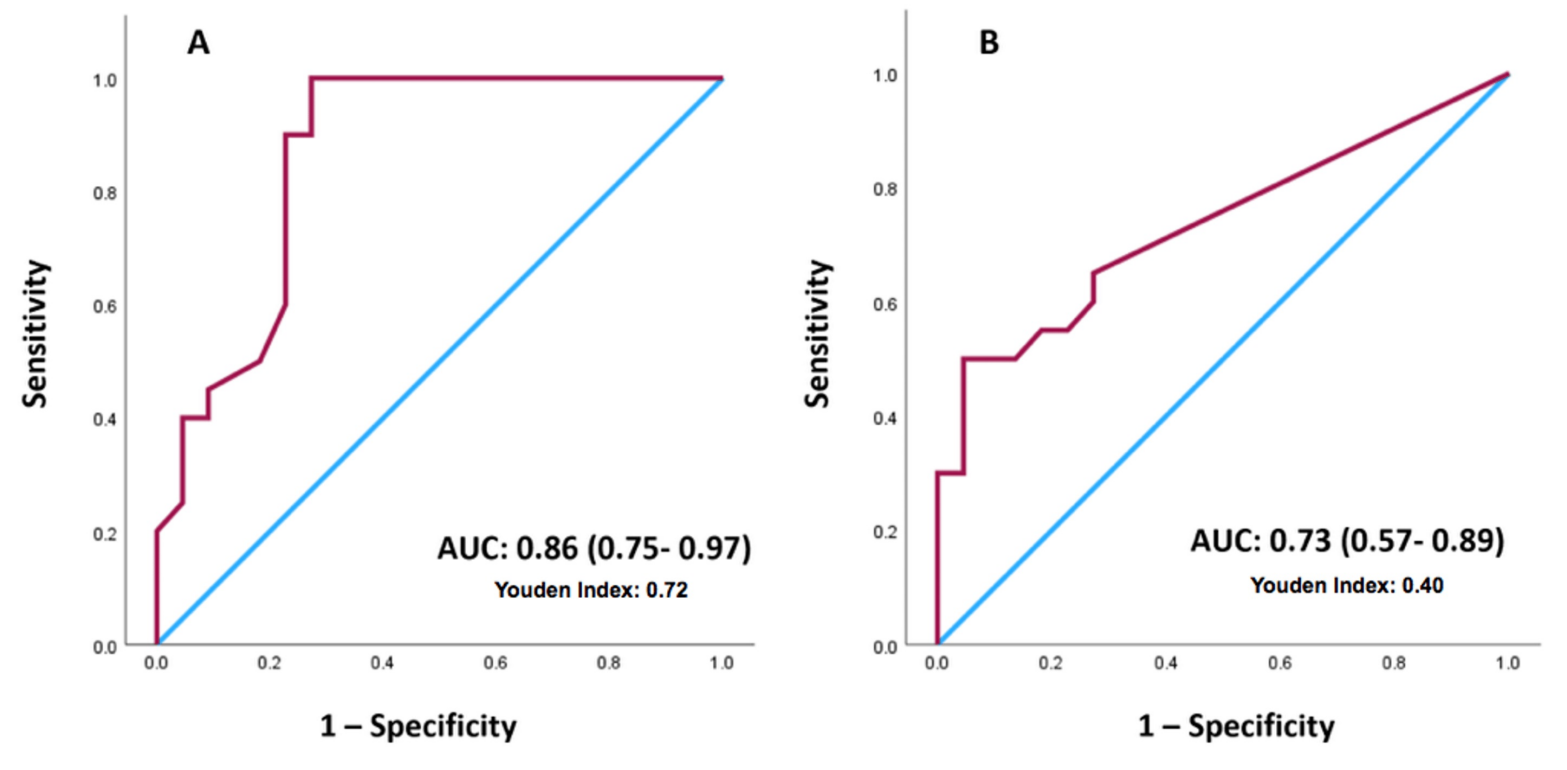
ROC curve for incomplete response vs. complete response - MRI (A) and PET (B) ROC curve: Receiver operating characteristic curve; AUC: Area under the ROC curve; PET: Positron emission tomography; MRI: Magnetic resonance imaging

ROC curve analysis and the maximum YI indicated that the cutoff PET maximum standardized uptake value (SUVmax) and MRI anteroposterior (AP) diameters are 2.25 and 10 mm, respectively (Figure 3).

**Figure 4:**
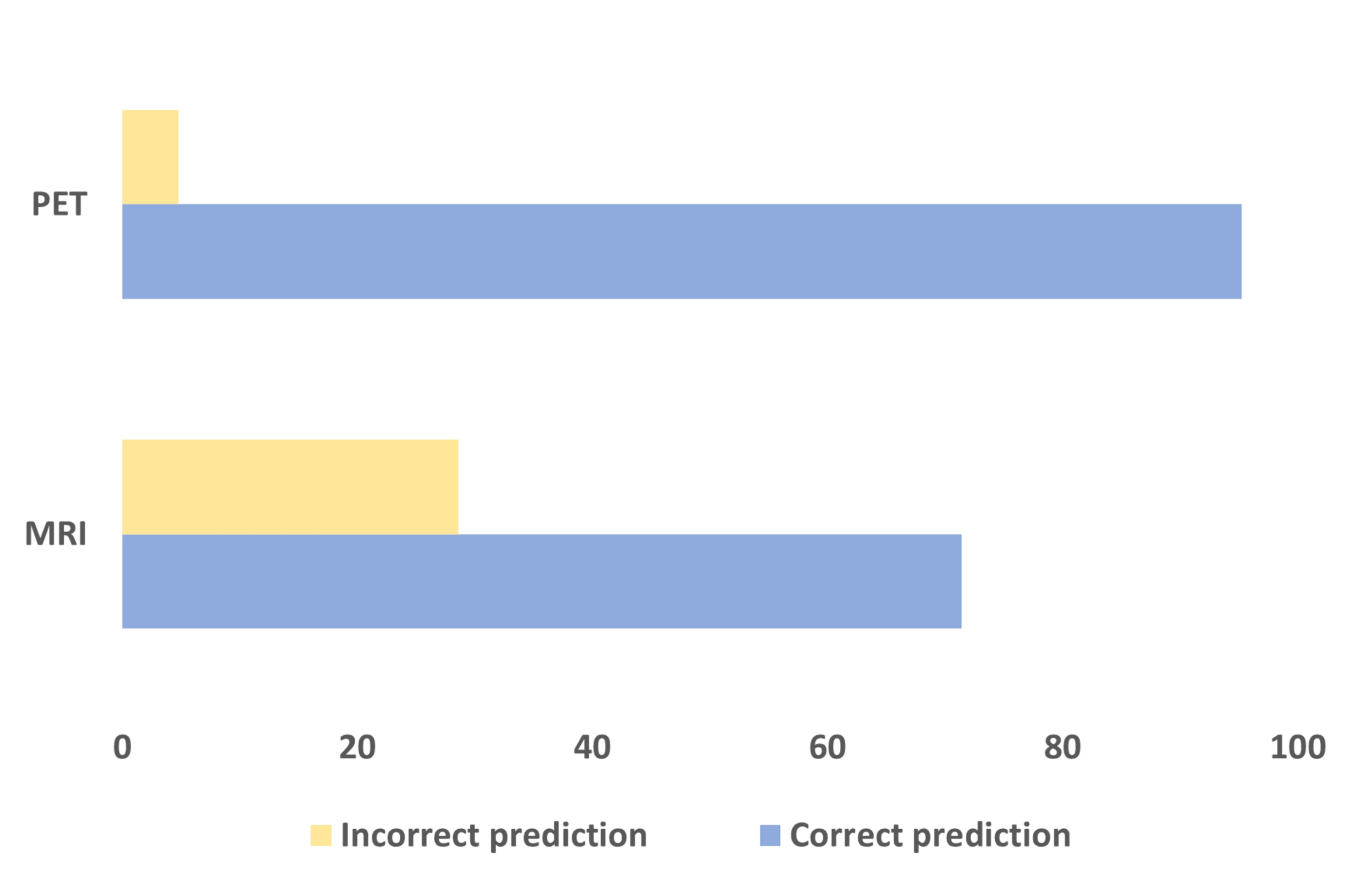
Accuracy of the pCR prediction model for PET and MRI PET showed 95% and MRI showed 71% accuracy of prediction for pCR. PET: Positron emission tomography; MRI: Magnetic resonance imaging; pCR: Pathological complete response

Using the prediction model, a confusion matrix was generated for the training data. The model indicated that the accuracy for predicting the pCR was 95.2% in PET vs. 71.4% in MRI datasets (Figure 4).

## Discussion

In the past, patients with locally advanced breast cancer (such as T3) would typically need to undergo radical mastectomy. However, the use of NAC has made it possible for most of these patients to have breast-conserving surgery instead by reducing the size of the tumor [2]. Predicting and determining pCR after the use of NAC is therefore essential in order to resort to less aggressive surgical resection and eventually lessen the overall morbidity [4]. Both MRI and PET/CT are useful investigations to predict pCR [12]. Our study assessed FDG PET/CT versus MRI in monitoring treatment responses to NAC in patients with breast cancer. The produced results will aid clinicians in choosing the right strategy for the assessment of pCR, modulating neoadjuvant therapy, and thereby gain more insights into the management of locally advanced breast cancer.

SUVmax is a widely evaluated parameter for the prediction of pCR using FDG PET/CT. According to a recent meta-analysis that included 1630 patients [13], SUVmax was successful in predicting pCR, and it also had the potential to be used to guide management in clinical practice [14]. Multiple studies suggested different cutoffs for SUVmax decrease to differentiate between pCR and non-pCR patients. A cutoff of 48.87% was set by Hulikal et al. with a sensitivity and specificity of 63.4 % and 75%, respectively [15]. Another study suggested optimal SUVmax decrease after the second cycle of chemotherapy with a value of 59.1%, positive predictive value of 50.0%, negative predictive value of 70.0%, and accuracy of 86.3% [16]. The other useful parameters to assess the tumor metabolism and prognosis by PET/CT are metabolic tumor volume (MTV) and total lesional glycolysis (TLG). While MTV signifies tumor burden by the actual size of the tumor tissue actively taking FDG, TLG denotes the median value of FDG uptake in that area [17]. However, these parameters have been found to be of more value in predicting the prognosis of other cancers like head and neck cancers, lung cancers, and uterine cancers. The standardized uptake value (SUV) or SUV corrected to lean body mass (SUL) remains the most important parameter in predicting prognosis in breast cancers [18]. According to PET response criteria in solid tumors (PERCIST) criteria, complete metabolic response is defined as complete normalization of all target and non-target lesions to SUL less than mean liver SUL, making it indistinguishable from surrounding normal tissue. Partial response is identified by a greater than 30% decrease or a minimum of 0.8 unit decrease in SUL peak [19]. In our study, out of the 65 patients who had complete pathological response post-neoadjuvant, 61 patients showed a complete response as per PERCIST criteria in PET/CT. Out of the four patients who had a partial response by PET/CT, the decrease in SUVmax was considerably significant (over 80%) but did not qualify as a complete resolution.

MRI has multiple parameters to predict pCR. Diffusion coefficient parameters in the form of quantitative and semiquantitative parameters were used by Liang et al. to predict pCR [20]. These parameters are associated with the rate at which contrast enhancement occurs, and they are influenced by the regional blood flow, vascular density, and vessel permeability. While some previous studies have shown that the rate at which the contrast diffuses from intravascular to extravascular space (K trans) is a significant predictor of pCR, Liang et al. found that differences in the rate of contrast enhancement (W-in, W-out) and the time taken to attain peak enhancement (TTP) could serve as important markers for early prediction of pCR. Surprisingly, the use of MRI enhancement as a predictor showed contradicting opinions. In a study by Gampenrieder et al., radiological complete response (rCR) corresponded to only 48% of pCR [21]. Some others used volumetric parameters such as the longitudinal diameter of the tumor before and after NAC to predict pCR. Lesion size too did not predict pCR in an accurate fashion [22]. These discrepancies were attributed to variations in the degree of angiogenesis in different tumor subtypes and distinctive shrinkage patterns. Our study showed rCR corresponding to pCR in 17 out of 65 patients (26.2%). This could be explained by the variations in tumor subtypes in our study. The majority of patients in our study had triple-negative and HER2-positive disease, both of which subtypes display excellent response to neoadjuvant therapy. The concept of MRI being more sensitive in determining the size and extent in the setting of substantial residual disease as compared to minimal or no residual disease as in our case is therefore validated by our study [23].

Several studies have evaluated and compared different modalities of imaging to predict pathological complete response post-neoadjuvant therapy. Cheng et al. performed a meta-analysis in 2012 and concluded that FDG PET/CT has a reasonable sensitivity in predicting response but relatively low specificity. In another meta-analysis comparing FDG PET with MRI, Sheikhabai et al. concluded that the overall diagnostic accuracy of MRI is better than FDG PET with a pooled sensitivity of 0.88 and a pooled specificity of 0.63 [12]. However, they made an interesting observation. FDG PET proved to be superior to MRI with a sensitivity of 0.91 and specificity of 0.69 when calculated in the intra-NAC setting. This could be due to a decrease in energy use of tumor cells that starts early on during neoadjuvant therapy. However, the downside is that NAC works by causing apoptosis, inflammation, and cell death. This can lead to a false positive FDG uptake in an otherwise completely responding tumor. Many studies have pointed to the superiority of MRI to FDG PET with a higher specificity for the former. The explanation for the increase in sensitivity of MRI in the later part of NAC is probably due to the fact that NAC almost always causes tumor shrinkage as part of the response. However, the actual shrinkage starts a few weeks (approximately two cycles) after initiation of NAC [24]. In an intra-NAC setting, therefore, MRI has limitations in assessing tumor response. One reason is that the enhancement of active tumor tissue and fibrosis with ongoing inflammation is difficult to tell apart [10]. The other significant influencing factor is the pattern of shrinkage that can alter MRI assessment.

Various studies have described and diversified tumor shrinkage patterns post-NAC as concentric and dendritic shrinkage according to MRI. Huang Y et al., in their study, scrutinized radiomics features and clinicopathological characteristics of tumors to predict tumor shrinkage patterns post-NAC [25]. They classified the shrinkage patterns as type I, which included the pCR (no residual) category and the concentric shrinkage pattern. Type II included dendritic shrinkage variants like diffuse shrinkage and multifocal shrinkage. This was classified in the poor shrinkage group as it makes some patients unfit for breast-conserving surgeries and leads to positive resection margins. These patients eventually undergo excision of margins or mastectomy. The concentric shrinkage pattern was observed commonly in triple-negative or HER2-positive tumors. Our study comprised predominantly such patients (42 HER2-positive and 19 triple-negative) who ended up having complete pathological responses. The majority of them showed concentric shrinkage patterns. This sheds light on the higher sensitivity in MRI post-NAC, which was probably due to the dendritic shrinkage pattern in a few patients.

We acknowledge the following limitations in our study: (a) The timing of MRI in the second week of the menstrual cycle was not possible in the study as we had to avoid delay in surgery. This could have interfered with tumor assessment due to background parenchymal enhancement. (b) Some authors include clearance of residual disease in axillary nodes as well [26]. Our study primarily focuses on the response to breast disease only. (c) The sample size and the follow-up period for monitoring and detecting recurrence were relatively low in our study. Only two of our study patients developed recurrence during the average follow-up period of three years. However, a larger study population and a longer follow-up period are necessary to validate our study findings. The differences in neoadjuvant regimens may have a bearing on the ability of different investigations to predict pCR. This factor was not taken into account in our study due to the small size of the cohort.

## Conclusions

Our study indicated that PET/CT is more sensitive in predicting pCR in post-neoadjuvant settings for breast cancer. On the other hand, when there is an incomplete response with significant residual disease, dynamic contrast-enhanced MRI serves as a better diagnostic performer. The combined use of PET/CT and MRI is highly beneficial in predicting pCR and achieving oncological clearance with less aggressive resection and reduced morbidity.
